# Pre-pandemic and pandemic healthcare utilisation among children with intellectual disabilities compared to the general population: a register study (IDcare)

**DOI:** 10.1136/bmjpo-2025-004298

**Published:** 2026-06-03

**Authors:** Anna Axmon, Magnus Sandberg

**Affiliations:** 1 Department of Laboratory Medicine, Lund University, Lund, Sweden; 2 Department of Health Sciences, Lund University, Lund, Sweden

**Keywords:** COVID-19

## Abstract

**Objective(s):**

To investigate utilisation patterns of different types of healthcare in a pre-pandemic and pandemic period, respectively, among children with intellectual disabilities in comparison to their age peers in the general population.

**Study design:**

Based on 8 years of register data, we identified 1947 children with intellectual disabilities (ID cohort) and 145 998 children from the general population (gPop cohort). All visits with physicians in outpatient care and all inpatient episodes during a pre-pandemic (2014–2019) and a pandemic (2020–2021) were identified.

**Results:**

During both periods, children in the ID cohort were more likely than those in the gPop cohort to have at least one healthcare visit (relative risk 1.06, 95% CI 1.01 to 1.11 during the pre-pandemic period and 1.31, 1.25 to 1.38 during the pandemic period). The largest effects were found for somatic inpatient care, particularly during the pandemic period. The opposite was found for psychiatric specialist care and primary care, where the effect was larger during the pre-pandemic period. Overall, there was a consistent pattern of increased relative rates of utilisation for unplanned care that was less evident for planned care.

**Conclusion(s):**

Although the results do not indicate any discrimination against children with intellectual disabilities during the COVID-19 pandemic, the results regarding unplanned care highlight the unmet healthcare needs in this group.

**WHAT IS ALREADY KNOWN ON THIS TOPIC**

- Children with intellectual disabilities have higher rates of ill health as well as high levels of healthcare utilisation. However, there are indications of unmet healthcare needs in this group.

**WHAT THIS STUDY ADDS**

- Even though children with intellectual disabilities have higher levels of planned healthcare than children in the general population, it is not enough to completely meet their healthcare needs. Even so, children with intellectual disabilities seemed to be a priority for healthcare services during the pandemic period.

**HOW THIS STUDY MIGHT AFFECT RESEARCH, PRACTICE OR POLICY**

- As unplanned healthcare represents a large proportion of the total healthcare use among children with intellectual disabilities, healthcare providers need to ensure they have enough capacity to provide such care for this group.

## Introduction

 Intellectual disability is characterised by limitations in cognitive functioning and adaptive behaviour. Compared with their age peers in the general population, children with intellectual disabilities have higher healthcare utilisation such as emergency department contacts and hospitalisations.^1^ This is to be expected as they have higher rates of chronic conditions such as epilepsy^2 3^ and asthma,^3^ as well as more mental health problems.^4^ Yet, despite their high healthcare utilisation, there are reports of unmet healthcare needs among children with intellectual disabilities.^5 6^ Unmet healthcare needs may stem from barriers to accessing healthcare, which is common among people with intellectual disabilities.^7 8^ However, for children, seeking healthcare relies more on the parent than the child. Thus, barriers such as communication difficulties should be a lesser concern in this group, as communication with healthcare professionals is the responsibility of the parent rather than the patient. Even so, seeking healthcare for a child with intellectual disabilities may differ from seeking healthcare for a child without. In a scoping review, parents of children with intellectual disabilities have testified to the need of working in a partner-like relationship with healthcare professionals and being more than a parent.^9^ To be able to support parents of children with intellectual disabilities, it is important to understand healthcare seeking patterns, including use of different types and levels of healthcare.

According to the Andersen model,^10^ healthcare utilisation is conceptualised as being determined by three main factors: healthcare need, access to services and health-seeking behaviour. Healthcare need reflects the underlying burden of disease and comorbidities, which is typically higher among children with intellectual disabilities.^2^,^4^ Access to services includes structural and organisational aspects such as availability of care, waiting times and financial barriers, whereas health-seeking behaviour relates to how and when caregivers decide to seek care for the child. In settings with universal healthcare systems and minimal financial barriers, such as Sweden, access to healthcare services for children is generally high. For example, participation in the Swedish national child health programme is close to 100%,^11 12^ reflecting a near-universal access to planned child healthcare services. However, high levels of access to healthcare services do not necessarily ensure that healthcare needs are adequately met. Children with intellectual disabilities may have complex and ongoing healthcare needs, and previous research has demonstrated that unmet healthcare needs can persist despite frequent use of healthcare services.^6^ Even so, the high access suggests that any changes in healthcare utilisation patterns are less likely to reflect baseline structural barriers and may instead indicate changes in healthcare availability or altered care-seeking behaviour among parents. Against this background, the COVID-19 pandemic provides a natural context in which both access to healthcare services and health-seeking behaviour may have been altered.

Studies from other western countries^13^,^15^ suggest that during the COVID-19 pandemic, children with intellectual disabilities experienced increased mental ill health and barriers to healthcare. Although Sweden took a different approach than the rest of the world during the pandemic, with no forced lock-down or closing of elementary schools, services for people with intellectual disabilities were reduced,^16^ and the high demand put on the healthcare system resulted in decreased surgeries and somatic inpatient care and fewer contacts with physicians in specialist outpatient care.^17^ We hypothesised that children with intellectual disabilities would maintain higher levels of healthcare utilisation than children in the general population during both periods, reflecting their greater healthcare needs. However, we further hypothesised that the relative differences between the groups could change during the pandemic period because of differential impacts on healthcare access and/or care-seeking behaviour. Thus, the aim of this study was to investigate utilisation patterns of different types of healthcare in a pre-pandemic and pandemic period, respectively, among children with intellectual disabilities in comparison to their age peers in the general population.

## Methods

### Study design and setting

This is a register-based cohort study comparing healthcare utilisation among children with intellectual disabilities to that of a same-aged referent cohort from the general population during a pre-pandemic (2014–2019) and pandemic (2020–2021) period. This study is set in Skåne, the southernmost region of Sweden, with almost 1.5 million inhabitants in 2024, corresponding to almost 15% of the total Swedish population. Here, as in the rest of Sweden, public healthcare is provided free of charge to people under the age of 20 years, as is privately organised care if the care provider is under contract with the county council. Child healthcare centres are organised within primary care and responsible for the national child health programme. The programme includes examinations by a physician at 4 weeks, 6 months, 12 months and 3 years of age, and participation is very high, close to 100%.^11 12^ Diagnoses of intellectual disabilities are made in psychiatric specialist care and often during childhood. It is common that autism spectrum disorder and intellectual disabilities are assessed simultaneously.

In Skåne, all healthcare contacts with public care providers or private care providers under contract with the county council are recorded in the Skåne Healthcare Register. For each healthcare contact, data on, for example, type of consultation (visit, phone or other), whether the patient was admitted through the emergency department (specialist care) or made the appointment the same day (primary care), the profession of the person treating (eg, physician or nurse), as well as one primary and several secondary diagnoses coded according to the International Statistical Classification of Diseases and Related Health Problems 10th Revision, are available.

### Study population

The IDcare project^18^ is based on all people residing in Skåne on 1 January 2014, who are divided into a cohort of people with intellectual disabilities (the ID cohort, n=14 716) and a referent cohort from the gPop (the gPop cohort, n=1 226 955). In the present study, we focused on children who were under 18 years old during the entire study period (2014–2021), that is, those born between 2004 and 2013. We excluded those who died in 2014, as we did not know the month and day of death and could therefore not determine if these had had a chance of contributing with any time in the study. Children from the ID cohort were only included if they had a diagnosis of intellectual disability (F70-F79 in ICD-10) or Down syndrome (Q90) during the study period. Thus, the ID cohort comprised 1 947 children (1221 boys and 726 girls) and the gPop cohort 145 998 children (74 496 boys and 71 502 girls).

### Statistics

We identified all outpatient healthcare visits (ie, physical contacts) where the care provider was a physician and all episodes in inpatient care. Each visit was categorised according to healthcare area (primary care, private care, somatic specialist care and psychiatric specialist care), and as inpatient or outpatient, and planned or unplanned. We determined the number of visits during each period (pre-pandemic and pandemic). Moreover, we determined the number of bed days from overnight stays in inpatient care among those with at least one inpatient episode. As the number of bed days was skewed to the left (ie, towards zero), we categorised this as low (below the median) or high (at or above the median). The cut-points used were two for somatic care during both periods, nine for psychiatric care pre-pandemic and seven for psychiatric care during the pandemic period.

All outcomes (having at least one healthcare visit, number of healthcare visits and number of bed days) in the ID cohort were compared with those in the gPop cohort using Poisson regression, thereby estimating relative risks (RRs) with 95% CIs. For dichotomous outcomes (having at least one healthcare visit and having high/low number of bed days), the RR should be interpreted as the RR of having the outcome among children in the ID cohort compared with the children in the gPop cohort. The number of visits is an ordinal variable, and here, the RR should be interpreted as the RR for a one-step increase in number of visits for the children in the ID cohort compared with the children in the gPop cohort.

Potential differences in RR for the pre-pandemic and pandemic periods were assessed by regression models including cohort (gPop and ID), period (pre-pandemic and pandemic) and the interaction term between cohort and period. All models were adjusted for age at inclusion (ie, in 2014, continuous variable), country of birth (categorical variable) and sex (dichotomous variable). Moreover, to account for loss of data due to death, time in the study was determined for each period and used to adjust all main analyses (ie, not in the analyses of interaction between cohort and period).

We performed sensitivity analyses to account for (a) inflation of numbers of contacts in psychiatric care due to assessment of intellectual disabilities and (b) primary care due to check-ups in the national child health programme. All data management and statistical analyses were performed using IBM SPSS Statistics 29 and 30. P values less than 0.05 were considered statistically significant. Results are only presented for groups comprising at least five people.

## Results

### Participant characteristics

Fifteen children (0.8%) in the ID cohort and 56 children (0.0%) in the gPop cohort died during the study period. Country of birth was categorised into four groups: born in Sweden to two parents born in Sweden (n=953, 49% in the ID cohort and n=88 546, 61% in the gPop cohort), born in Sweden to one parent born in Sweden and one born abroad (n=253, 13% and n=20 298, 14%), born in Sweden to two parents born abroad (n=588, 30% and n=28 370, 19%) and born abroad (n=153, 8% and n=8 784, 6%). Among the children in the ID cohort, 132 (7%) had diagnosis of both intellectual disability and Down syndrome, 1 779 (91%) of intellectual disability only and 36 (2%) of Down syndrome only. Among those with a diagnosis of intellectual disability, 1093 (57%) had mild intellectual disability (F70), 374 (20%) had moderate intellectual disability (F71), 137 (7%) had severe intellectual disability (F72), 43 (2%) had profound intellectual disability (F73) and 264 (14%) had other/unknown intellectual disability (F78/F79).

### Overall healthcare visits

**Figure 1:**
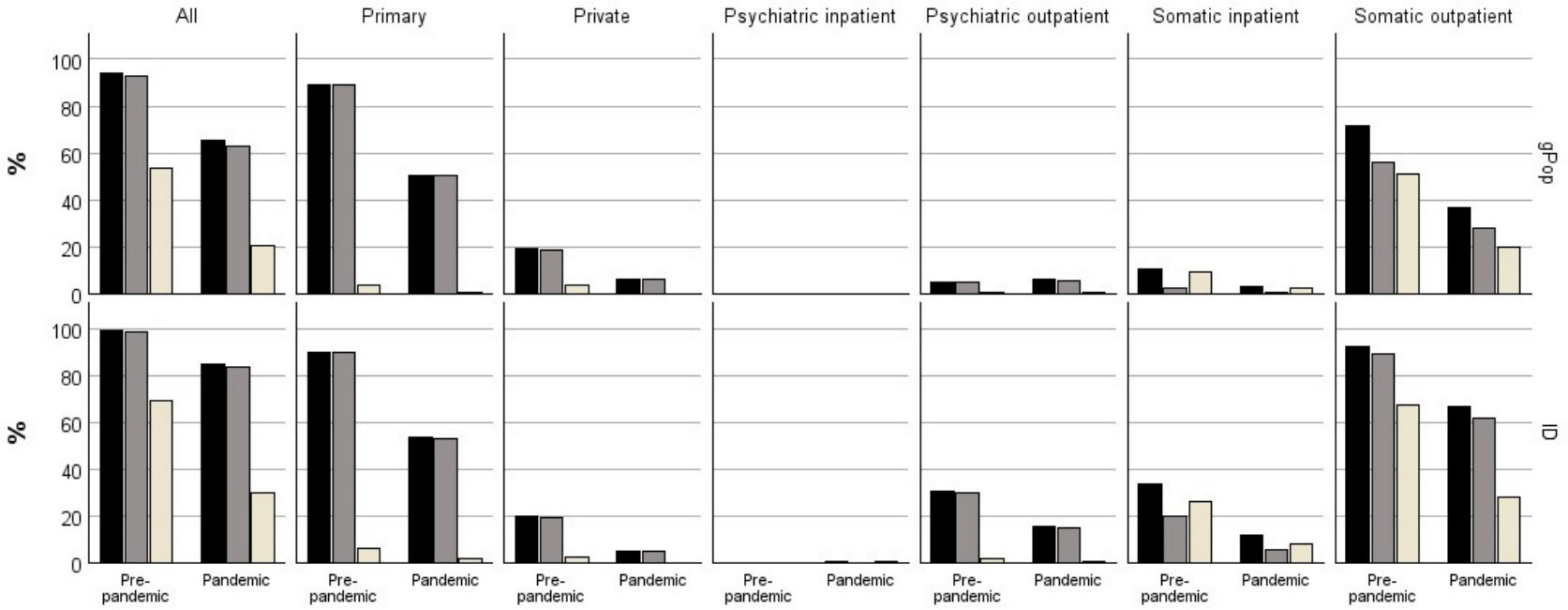
Percentage of 1947 children with intellectual disabilities (ID) and a referent cohort of 145 998 children from the general population (gPop) with at least one healthcare visit in different healthcare areas during a pre-pandemic (2014–2019) and a pre-pandemic (2020–2021) period (black bars=all visits, brown bars=planned visits, beige bars=unplanned visits).

**Figure 2:**
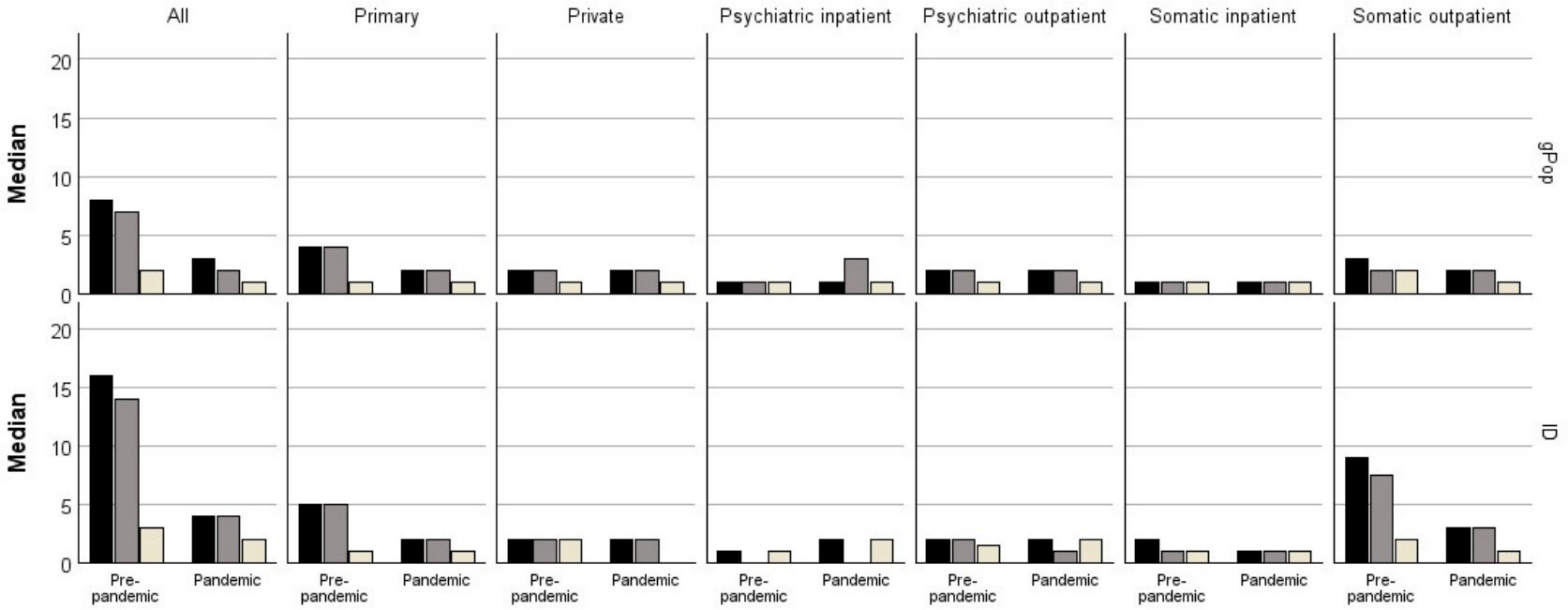
Median number of visits in different healthcare areas during a pre-pandemic (2014–2019) and a pre-pandemic (2020–2021) period among those with at least one among 1947 children with intellectual disabilities (ID) and a referent cohort of 145 998 children from the general population (gPop) during a pre-pandemic (2014–2019) and a pandemic (2020–2021) period (black bars=all visits, brown bars=planned visits, beige bars=unplanned visits).

**Figure 3:**
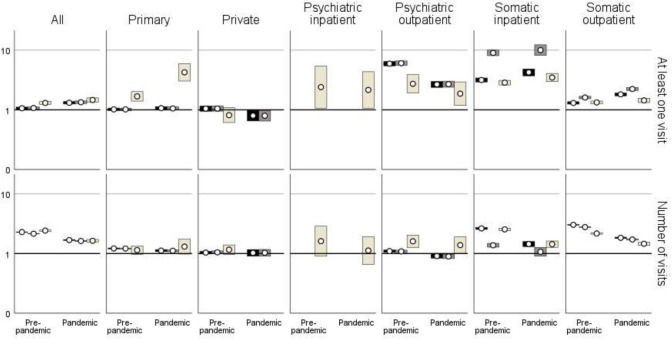
Relative risks (circles) with 95% CIs of having at least one healthcare visit (top) and number of visits (bottom) among 1947 children with intellectual disabilities (ID) compared with a referent cohort of 145 998 children from the general population (gPop) (black bars=all visits, brown bars=planned visits, beige bars=unplanned visits).

During the pre-pandemic period, 99% of the children in the ID cohort and 94% of the children in the gPop cohort had at least one healthcare visit (figure 1; online supplemental file 1). The median number of visits among those with at least one was 16 in the ID cohort and eight in the gPop cohort (figure 2; online supplemental file 1), corresponding to yearly averages of 2.7 and 1.3, respectively. During the pandemic period, the percentage of children with at least one visit decreased to 85% in the ID cohort and 65% in the gPop cohort (figure 1; online supplemental file 1), and the median number of visits decreased to 4 (yearly average 2.0) and 3 (yearly average 1.5), respectively (figure 2; online supplemental file 1). This corresponds to a 6% higher likelihood of having at least one healthcare visit during the pre-pandemic period and a 31% higher likelihood during the pandemic period for the ID cohort, when adjusting for year of birth, country of birth and sex (figure 3; online supplemental file 1). The higher relative difference associated with the ID cohort during the pandemic period (p for interaction cohort and period<0.001) was primarily driven by a larger decrease in percentage in the gPop cohort. In contrast, the increased likelihood associated with the ID cohort for number of visits decreased during the pandemic period (p<0.001), due to a larger decrease in number of visits in the ID cohort.

When assessing planned and unplanned healthcare separately, similar patterns were found as for total healthcare. Thus, increased likelihood for the ID cohort for both periods, with the largest increase for having at least one healthcare visit during the pandemic period (p for interaction<0.001 for planned healthcare and p=0.009 for unplanned healthcare) and the largest increase for lower number of healthcare visits during the pre-pandemic period (p<0.001 for both planned and unplanned healthcare).

### Healthcare visits by healthcare area

Stratifying by healthcare areas in both inpatient and outpatient care, all areas but private care displayed a pattern of higher likelihoods during both periods for both having at least one visit and number of visits associated with the ID cohort (figures1^3^; online supplemental file 1). In all types of specialist care, the increased likelihood for having at least one visit was highest for planned visits (except for psychiatric inpatient care, where planned visits were rare and therefore not evaluated), whereas in primary care, the increased likelihood associated with the ID cohort was most evident for unplanned visits. Although a similar pattern was found for number of visits to somatic outpatient care, that is, with the largest relative difference for planned care, the number of unplanned healthcare visits carried the largest relative difference in psychiatric outpatient and somatic inpatient care. The likelihood of having at least one visit in primary care and somatic inpatient and outpatient care associated with the ID cohort tended to be higher during the pandemic period than during the pre-pandemic period. The opposite was found for number of visits, where the increased likelihood associated with the ID cohort was lower during the pandemic period. For psychiatric specialist care, the increased likelihood associated with the ID cohort was lower during the pandemic period, both for having at least one visit and number of visits.

### Bed days in inpatient care

During the pre-pandemic period, 458 children (75%) in the ID cohort and 7011 children (52%) in the gPop cohort had at or above the median of bed days in somatic inpatient care (RR 1.41, 95% CI 1.28 to 1.55). The corresponding numbers for the pandemic period were 156 children (74%) in the ID cohort and 1738 children (53%) in the gPop cohort (RR 1.37, 95% CI 1.16 to 1.61, p for interaction 0.761). For psychiatric inpatient care, the number of children in the ID cohort in each group (below and at or above the median) was less than five, and therefore, we present no results for this.

### Sensitivity analyses

When excluding 299 of 6339 planned visits in psychiatric outpatient care where the primary diagnosis was intellectual disability or the primary diagnosis was autism spectrum disorder and intellectual disability was included among the secondary diagnoses, the risk estimates remained similar for having at least one visit (RR 6.04 vs 5.68 for the pre-pandemic period and 2.69 vs 2.26 for the pandemic period) and number of visits (RR 1.09 vs 1.04 for the pre-pandemic period and 0.89 vs 0.87 for the pandemic period).

Sensitivity analyses excluding planned visits in primary care with child healthcare centre physicians or any of the listed diagnoses Z00.1 (routine child examination; 837 of 19 850 visits in the ID cohort and 55 544 of 897 405 visits in the gPop cohort) did not have any major impact on the results for either having at least one visit (RR 1.02 vs 1.02 for the pre-pandemic period and 1.05 vs 1.06 for the pandemic period) or for number of visits (1.22 vs 1.22 for pre-pandemic period and 1.11 vs 1.11 for the pandemic period).

## Discussion

We found a pattern of higher healthcare utilisation among children with intellectual disabilities than children in the general population in specialist care, psychiatric as well as somatic and inpatient as well as outpatient. The pattern was consistent for both planned and unplanned healthcare and during both the pre-pandemic and pandemic period. However, in primary care, a higher likelihood was found only for unplanned care. Among children with at least one specialist care visit, children with intellectual disabilities had more visits than the children in the general population. This was particularly evident during the pre-pandemic period.

Before considering implications of the results, some limitations of the study need to be acknowledged. The most important one is the ageing of the children included. As all children were included at the same time, they aged the same during the study period. Although we attempted to account for this by adjusting all analyses for age at inclusion, we cannot rule out that the differences in effect found for the two different periods are due to a change in health and disease patterns as the children age rather than to the pandemic. This is supported by previous findings that the higher likelihood of health services use among 9-year-olds with intellectual disabilities is not maintained at age 13.^6^ The period differences found in the present study may also be related to reduced access to healthcare when transitioning into adult care, as previous studies have identified gaps in service provision and challenges in continuity of care during this transition.^19^ Future research should focus on assessing changes in healthcare utilisation during such transition while considering simultaneous effects of the COVID-19 pandemic.

Another limitation of the present study is the lack of data on parental socioeconomic variables, such as income and educational level. Previous studies in the general population present mixed results on the impact of parental socioeconomy on the child’s healthcare utilisation. Whereas low status has been associated with more inpatient treatments^20^ and higher healthcare costs,^21^ high status has been linked to higher vaccination uptake^22 23^ and more specialised care for chronic pain^24^ and diabetes.^25^ Meanwhile, others have found no effect on access to specialised mental health services^26^ or outpatient hospital services.^20^ Even so, we cannot rule out that socioeconomic status may have an impact on the measures of healthcare utilisation used in the present study, particularly as parental socioeconomic status has also been linked to the prevalence of intellectual disability in the child.^27^ However, due to the inconsistency in previous results, we are unable to speculate in which direction our results may be skewed. Future studies should aim at including parental sociodemographic factors at potential pathways to healthcare utilisation among children with intellectual disabilities.

Having considered the limitations of the study, we would also like to highlight its major strengths. These include the use of national and regional registers to establish the cohorts as well as determine outcomes and potential confounders. The registers used are maintained by national and regional authorities, and participation is not optional. Thus, the risks of selection bias and misclassification of outcomes are minimal. Further strengths are the inclusion of all children in a geographical area covering about 15% of the entire Swedish population and the long study period, which covers both time before and during the COVID-19 pandemic. Given the large coverage in population and years, we believe the results from this study are generalisable to other countries with the similar welfare and healthcare systems as Sweden, including most Western countries and particularly countries within the European Union.

Previous reports on healthcare utilisation among children with intellectual disabilities are scarce. However, the results from our study align well with the few existing studies. Our findings of higher likelihoods for inpatient care (ie, hospitalisation) across all types of care are supported by previous studies, which also found higher likelihoods of both planned (elective) and unplanned (emergency) hospital admissions among children and young people with intellectual disabilities,^28 29^ as well as higher likelihood of emergency department visits.^30 31^ Moreover, in agreement with previous research,^6^ we did not find any higher likelihood of having at least one planned visit in primary care during either period.

When interpreting the findings from the present study, it is important to consider that changes in healthcare utilisation may reflect changes in healthcare need, access to healthcare services and health-seeking behaviour.^10^ Reduced availability of services, for example, due to postponed or cancelled care, represents a system-level barrier, whereas changes in healthcare seeking behaviour (reflected in this study by parental decisions to seek care) may reflect behavioural responses, such as avoiding healthcare contacts during the pandemic. With this in consideration, we suggest two major implications of our findings. The first is the indication of unmet healthcare needs among children with intellectual disabilities suggested by the higher likelihood of unplanned healthcare. In comparison with children from the general population, children with intellectual disabilities have worse overall health and larger number of co-morbidities.^2 4 32^ Hence, it is only to be expected that they have higher levels of planned healthcare. However, unplanned healthcare has been suggested to be an indicator of unmet healthcare needs and barriers to healthcare in different patient groups (eg,^33^,^35^). Thus, the higher likelihood for unplanned healthcare among children with intellectual disabilities found in the present study should be cause for concern as, regardless of the driver behind these results, whether they be system-level barriers or parental behavioural responses, it may reflect limited availability of planned care leading to increased reliance on unplanned services. This is particularly true for somatic specialist care, where there was not only an increased likelihood compared with the gPop cohort but also a high prevalence of children in the ID cohort with at least one visit. Almost two-thirds of the children in the ID cohort had unplanned visits in outpatient care, and almost a fourth were hospitalised during the pre-pandemic period. Children who have been hospitalised have been found to have higher likelihood for later mental health diagnoses,^36 37^ and thus, a reduced need for hospitalisations should be an aim for healthcare services. Even though children with intellectual disabilities have more complex health, we found no differences in planned primary care visits between the cohorts. This suggests that improvements regarding preventive care may be warranted. Moreover, as primary care is intended as first line for healthcare for preventable diseases as well as responsible for long-term management of chronic diseases, an increase in planned primary care visits could potentially meet some of the needs that currently cause unplanned visits. Following the reasonings above, suggestions for pathways to reduce unplanned healthcare among children with intellectual disabilities may include preventive care, health check-ups and increased frequency of planned healthcare for known disorders, particularly in primary care. Future studies should focus on further investigating these, and other, pathways to reduce unmet healthcare needs among children with intellectual disabilities. An important clinical implication of the results from this study is that until such pathways have been identified and implemented, healthcare providers need to ensure they have enough capacity to provide unplanned healthcare to children with intellectual disabilities, as such care represents a large proportion of their total healthcare use. Considering the higher and more complex disease burden among children with intellectual disabilities, this is particularly important in somatic specialist care.

The second implication of the results from the present study is a positive one, namely, that we found few indications of discrimination against children with intellectual disabilities in primary or somatic specialist care during the pandemic. The only potential sign of such discrimination was the reduction in number of visits among those with at least one in the ID cohort parallel to an increased number in the gPop cohort. The larger decrease in healthcare utilisation observed in the gPop compared with the ID cohort may suggest that health-seeking behaviour changed differently among parents of children with and without intellectual disabilities. In contrast, the smaller decrease in the ID cohort may indicate that healthcare needs in this group necessitated continued contact with healthcare services, or that these children were prioritised within the healthcare system. Previous studies have found that the healthcare provision for children in vulnerable groups was negatively impacted by the pandemic. For example, clinically vulnerable children had higher reductions in hospital care than their age peers,^38^ and children with motor impairments had reduced access to paediatric rehabilitation.^39^ This means that barriers to healthcare could occur at different stages. One stage may be in entering the healthcare system whereas another may be to have your needs met once you are in the system. Viewing access to healthcare this way, our finding suggests reduced access for those that entered the healthcare system. Another possible indication of discrimination was that the increased likelihood of psychiatric care use associated with the ID cohort, although consistently high, was halved during the pandemic period. This was primarily driven by a large reduction in the number of children in the ID cohort with at least one healthcare visit. We investigated whether healthcare visits for assessment of intellectual disabilities during the early childhood years could be a driver for the reduction in psychiatric healthcare visits during the later part of the study period. However, the data provided no evidence of such a pathway. Thus, a remaining explanation may be that children with intellectual disabilities and mental health problems did not receive appropriate level of psychiatric care during the pandemic. This is in agreement with previous studies, which have found that children with special healthcare needs experienced increased odds of unmet mental healthcare needs during the pandemic.^40^ Therefore, an important clinical implication of our results is the need for healthcare providers to ensure that there are strategies in place to secure continuity of care and sufficient support even during times when the healthcare system faces unexpected challenges such as the COVID-19 pandemic. Overall, our findings highlight the importance of interpreting healthcare utilisation patterns in the context of both healthcare system factors and care-seeking behaviour, particularly during periods of disruption such as the COVID-19 pandemic.

In summation, we found patterns of increased public healthcare use among children with intellectual disabilities compared with their age peers in the general population. The pattern was most evident in specialist care and for unplanned healthcare visits and evident both prior to and during the COVID-19 pandemic. Although the results do not indicate any discrimination against children with intellectual disabilities during the COVID-19 pandemic, the study again highlights the unmet healthcare needs in this group.

## Supplementary material

10.1136/bmjpo-2025-004298 online supplemental file 1

## Data Availability

Data may be obtained from a third party and are not publicly available.
