# Supplementary material for: Pre-pandemic and pandemic healthcare utilisation among children with intellectual disabilities compared to the general population: a register study (IDcare)
Source: BMJ Paediatr Open. 2026 Jun 3;10(1):e004298. doi: 10.1136/bmjpo-2025-004298 (PMC13239560; doi:10.1136/bmjpo-2025-004298)
Supplement: online supplemental file 1 [file bmjpo-10-1-s001.docx]

|  |  |  |  |  |  |  |  | At least one visit | | | | | | | | Number of visits | | | | | | | |
| --- | --- | --- | --- | --- | --- | --- | --- | --- | --- | --- | --- | --- | --- | --- | --- | --- | --- | --- | --- | --- | --- | --- | --- |
|  |  | gPop | | | ID | | | Crude | | | | Adjusted | | | | Crude | | | | Adjusted | | | |
|  |  | n | % | M | n | % | M | RR | 95 CI | | p | RR | 95 CI | | p | RR | 95 CI | | p | RR | 95 CI | | p |
| **All** |  |  |  |  |  |  |  |  |  |  |  |  |  |  |  |  |  |  |  |  |  |  |  |
| All | P1 | 137 140 | 93.9 | 8 | 1 926 | 98.9 | 16 | **1.05** | **1.01** | **1.10** | **<0.001** | **1.06** | **1.01** | **1.11** | **<0.001** | **2.28** | **2.26** | **2.30** | **<0.001** | **2.29** | **2.27** | **2.31** | **<0.001** |
|  | P2 | 95 180 | 65.2 | 3 | 1 647 | 85.1 | 4 | **1.30** | **1.24** | **1.37** |  | **1.31** | **1.25** | **1.38** |  | **1.69** | **1.66** | **1.72** |  | **1.68** | **1.65** | **1.72** |  |
| Primary | P1 | 130 183 | 89.2 | 4 | 1 750 | 89.9 | 5 | 1.01 | 0.96 | 1.06 | 0.196 | 1.02 | 0.97 | 1.07 | 0.203 | **1.19** | **1.17** | **1.21** | **0.043** | **1.22** | **1.20** | **1.24** | **0.021** |
|  | P2 | 73 988 | 50.7 | 2 | 1 041 | 53.8 | 2 | 1.06 | 1.00 | 1.13 |  | **1.07** | **1.00** | **1.13** |  | **1.14** | **1.09** | **1.18** |  | **1.12** | **1.08** | **1.17** |  |
| Private | P1 | 28 359 | 19.4 | 2 | 392 | 20.1 | 2 | 1.04 | 0.94 | 1.15 | **0.013** | 1.05 | 0.95 | 1.16 | **0.012** | 1.01 | 0.96 | 1.06 | 0.907 | 1.04 | 0.99 | 1.09 | 0.713 |
|  | P2 | 9 029 | 6.2 | 2 | 93 | 4.8 | 2 | **0.78** | **0.63** | **0.95** |  | **0.80** | **0.65** | **0.98** |  | 1.01 | 0.89 | 1.15 |  | 1.03 | 0.90 | 1.17 |  |
| Psychiatric inpatient | P1 | 202 | 0.1 | 1 | <5 | 0.3 | 1 |  |  |  |  |  |  |  |  |  |  |  |  |  |  |  |  |
|  | P2 | 305 | 0.2 | 1 | <5 | 0.4 | 2 |  |  |  |  |  |  |  |  |  |  |  |  |  |  |  |  |
| Psychiatric outpatient | P1 | 7 122 | 4.9 | 2 | 592 | 30.4 | 2 | **6.23** | **5.73** | **6.78** | **<0.001** | **5.93** | **5.45** | **6.45** | **<0.001** | **0.93** | **0.89** | **0.97** | **0.044** | **1.10** | **1.05** | **1.15** | **<0.001** |
|  | P2 | 8 551 | 5.9 | 2 | 297 | 15.3 | 2 | **2.62** | **2.33** | **2.94** |  | **2.67** | **2.37** | **2.99** |  | **0.84** | **0.78** | **0.91** |  | 0.91 | 0.84 | 0.98 |  |
| Somatic inpatient | P1 | 15 471 | 10.6 | 1 | 658 | 33.8 | 2 | **3.19** | **2.95** | **3.45** | **<0.001** | **3.16** | **2.92** | **3.42** | **<0.001** | **2.76** | **2.65** | **2.87** | **<0.001** | **2.64** | **2.53** | **2.75** | **<0.001** |
|  | P2 | 3 959 | 2.7 | 1 | 231 | 11.9 | 1 | **4.40** | **3.85** | **5.02** |  | **4.24** | **3.71** | **4.85** |  | **1.47** | **1.33** | **1.61** |  | **1.44** | **1.31** | **1.59** |  |
| Somatic outpatient | P1 | 104 299 | 71.4 | 3 | 1 802 | 92.6 | 9 | **1.30** | **1.24** | **1.36** | **<0.001** | **1.30** | **1.24** | **1.37** | **<0.001** | **3.09** | **3.05** | **3.12** | **<0.001** | **3.03** | **3.00** | **3.07** | **<0.001** |
|  | P2 | 53 915 | 36.9 | 2 | 1 294 | 66.8 | 3 | **1.81** | **1.71** | **1.91** |  | **1.81** | **1.72** | **1.92** |  | **1.84** | **1.80** | **1.89** |  | **1.83** | **1.79** | **1.88** |  |
| **Planned** |  |  |  |  |  |  |  |  |  |  |  |  |  |  |  |  |  |  |  |  |  |  |  |
| All | P1 | 135 561 | 92.9 | 7 | 1 921 | 98.7 | 14 | **1.06** | **1.02** | **1.11** | **<0.001** | **1.07** | **1.02** | **1.12** | **<0.001** | **2.13** | **2.11** | **2.16** | **<0.001** | **2.15** | **2.13** | **2.17** | **<0.001** |
|  | P2 | 91 374 | 62.6 | 2 | 1 615 | 83.4 | 4 | **1.33** | **1.27** | **1.40** |  | **1.34** | **1.28** | **1.41** |  | **1.63** | **1.59** | **1.66** |  | **1.62** | **1.59** | **1.66** |  |
| Primary | P1 | 130 056 | 89.1 | 4 | 1 744 | 89.6 | 5 | 1.01 | 0.96 | 1.05 | 0.276 | 1.02 | 0.97 | 1.07 | 0.285 | **1.19** | **1.17** | **1.21** | **0.029** | **1.22** | **1.19** | **1.24** | **0.012** |
|  | P2 | 73 891 | 50.6 | 2 | 1 029 | 53.2 | 2 | 1.05 | 0.99 | 1.12 |  | 1.05 | 0.99 | 1.12 |  | **1.13** | **1.09** | **1.18** |  | **1.11** | **1.07** | **1.16** |  |
| Private | P1 | 27 478 | 18.8 | 2 | 382 | 19.6 | 2 | 1.04 | 0.94 | 1.15 | **0.011** | 1.05 | 0.95 | 1.16 | **0.010** | 1.02 | 0.97 | 1.08 | 0.948 | 1.05 | 1.00 | 1.11 | 0.902 |
|  | P2 | 8 951 | 6.1 | 2 | 92 | 4.8 | 2 | 0.77 | 0.63 | 0.95 |  | **0.80** | **0.65** | **0.98** |  | 1.02 | 0.90 | 1.16 |  | 1.03 | 0.91 | 1.17 |  |
| Psychiatric inpatient | P1 | 22 | 0.0 | 1 | <5 | 0.0 |  |  |  |  |  |  |  |  |  |  |  |  |  |  |  |  |  |
|  | P2 | 23 | 0.0 | 3 | <5 | 0.0 |  |  |  |  |  |  |  |  |  |  |  |  |  |  |  |  |  |
| Psychiatric outpatient | P1 | 6 915 | 4.7 | 2 | 584 | 30.0 | 2 | **6.33** | **5.82** | **6.89** | **<0.001** | **6.04** | **5.55** | **6.58** | **<0.001** | **0.92** | **0.88** | **0.97** | **0.035** | **1.09** | **1.04** | **1.14** | **<0.001** |
|  | P2 | 8 407 | 5.8 | 2 | 294 | 15.2 | 1 | **2.64** | **2.35** | **2.96** |  | **2.69** | **2.39** | **3.02** |  | **0.83** | **0.77** | **0.91** |  | **0.89** | **0.82** | **0.97** |  |
| Somatic inpatient | P1 | 3 077 | 2.1 | 1 | 385 | 19.8 | 1 | **9.38** | **8.44** | **10.43** | 0.338 | **8.98** | **8.06** | **10.01** | 0.201 | **1.41** | **1.31** | **1.51** | **0.003** | **1.38** | **1.28** | **1.48** | **0.004** |
|  | P2 | 769 | 0.5 | 1 | 107 | 5.5 | 1 | **10.49** | **8.57** | **12.84** |  | **10.01** | **8.15** | **12.29** |  | 1.06 | 0.90 | 1.26 |  | 1.07 | 0.90 | 1.27 |  |
| Somatic outpatient | P1 | 81 607 | 55.9 | 2 | 1 736 | 89.2 | 8 | **1.60** | **1.52** | **1.67** | **<0.001** | **1.61** | **1.53** | **1.68** | **<0.001** | **2.84** | **2.80** | **2.88** | **<0.001** | **2.77** | **2.74** | **2.81** | **<0.001** |
|  | P2 | 40 720 | 27.9 | 2 | 1 198 | 61.9 | 3 | **2.22** | **2.09** | **2.35** |  | **2.23** | **2.11** | **2.36** |  | **1.73** | **1.68** | **1.78** |  | **1.72** | **1.67** | **1.77** |  |
| **Unplanned** | |  |  |  |  |  |  |  |  |  |  |  |  |  |  |  |  |  |  |  |  |  |  |
| All | P1 | 78 099 | 53.5 | 2 | 1 353 | 69.5 | 3 | **1.30** | **1.23** | **1.37** | **0.011** | **1.30** | **1.24** | **1.38** | **0.009** | **2.45** | **2.40** | **2.51** | **<0.001** | **2.42** | **2.37** | **2.48** | **<0.001** |
|  | P2 | 29 538 | 20.2 | 1 | 578 | 29.9 | 2 | **1.48** | **1.36** | **1.60** |  | **1.46** | **1.35** | **1.59** |  | **1.64** | **1.56** | **1.73** |  | **1.64** | **1.56** | **1.72** |  |
| Primary | P1 | 5 139 | 3.5 | 1 | 118 | 6.1 | 1 | **1.72** | **1.43** | **2.07** | **<0.001** | **1.68** | **1.40** | **2.02** | **<0.001** | 1.15 | 0.98 | 1.35 | 0.449 | 1.14 | 0.97 | 1.34 | 0.385 |
|  | P2 | 573 | 0.4 | 1 | 37 | 1.9 | 1 | **4.87** | **3.49** | **6.79** |  | **4.25** | **3.04** | **5.94** |  | 1.31 | 0.98 | 1.74 |  | 1.31 | 0.98 | 1.74 |  |
| Private | P1 | 4 968 | 3.4 | 1 | 48 | 2.5 | 2 | **0.72** | **0.55** | **0.96** | 0.627 | 0.81 | 0.61 | 1.08 | 0.624 | 1.14 | 0.95 | 1.37 | 0.837 | 1.16 | 0.97 | 1.39 | 0.925 |
|  | P2 | 296 | 0.2 | 1 | <5 | 0.1 | 2 |  |  |  |  |  |  |  |  |  |  |  |  |  |  |  |  |
| Psychiatric inpatient | P1 | 188 | 0.1 | 1 | 6 | 0.3 | 1 | **2.39** | **1.06** | **5.39** | 0.793 | **2.40** | **1.06** | **5.41** | 0.786 | 1.43 | 0.82 | 2.50 | 0.372 | 1.61 | 0.91 | 2.87 | 0.302 |
|  | P2 | 291 | 0.2 | 1 | 8 | 0.4 | 2 | **2.07** | **1.03** | **4.18** |  | **2.15** | **1.07** | **4.35** |  | 1.02 | 0.61 | 1.70 |  | 1.12 | 0.66 | 1.92 |  |
| Psychiatric outpatient | P1 | 824 | 0.6 | 1 | 32 | 1.6 | 2 | **2.91** | **2.05** | **4.15** | 0.118 | **2.73** | **1.92** | **3.89** | 0.116 | **1.54** | **1.21** | **1.94** | 0.387 | **1.60** | **1.26** | **2.03** | 0.376 |
|  | P2 | 779 | 0.5 | 1 | 19 | 1.0 | 2 | **1.84** | **1.17** | **2.90** |  | **1.86** | **1.18** | **2.94** |  | 1.29 | 0.94 | 1.77 |  | **1.38** | **1.01** | **1.91** |  |
| Somatic inpatient | P1 | 13 427 | 9.2 | 1 | 516 | 26.5 | 1 | **2.88** | **2.64** | **3.15** | **0.016** | **2.85** | **2.61** | **3.11** | **0.008** | **2.64** | **2.51** | **2.77** | **<0.001** | **2.54** | **2.42** | **2.67** | **<0.001** |
|  | P2 | 3 374 | 2.3 | 1 | 161 | 8.3 | 1 | **3.60** | **3.07** | **4.21** |  | **3.47** | **2.96** | **4.07** |  | **1.45** | **1.29** | **1.64** |  | **1.44** | **1.27** | **1.62** |  |
| Somatic outpatient | P1 | 74 096 | 50.8 | 2 | 1 311 | 67.3 | 2 | **1.33** | **1.26** | **1.40** | 0.088 | **1.33** | **1.26** | **1.41** | 0.077 | **2.19** | **2.14** | **2.24** | **<0.001** | **2.17** | **2.11** | **2.22** | **<0.001** |
|  | P2 | 28 529 | 19.5 | 1 | 548 | 28.3 | 1 | **1.45** | **1.33** | **1.58** |  | **1.44** | **1.32** | **1.56** |  | **1.46** | **1.38** | **1.55** |  | **1.46** | **1.38** | **1.55** |  |
