# Supplementary material for: Pre-pandemic and pandemic healthcare utilisation among children with intellectual disabilities compared to the general population: a register study (IDcare)
Source: BMJ Paediatr Open. 2026 Jun 3;10(1):e004298. doi: 10.1136/bmjpo-2025-004298 (PMC13239560; doi:10.1136/bmjpo-2025-004298)
Supplement: Supplementary file 2 [file bmjpo-2025-004298.reviewer_comments.pdf]

## PEER REVIEW HISTORY

BMJ Paediatrics Open publishes all reviews undertaken for accepted manuscripts. Reviewers are asked to complete a checklist review form and are provided with free text boxes to elaborate on their assessment. These free text comments are reproduced below.

## ARTICLE DETAILS

| | |
|---|---|
| **TITLE (PROVISIONAL)** | Pre-pandemic and pandemic healthcare utilization among children with intellectual disabilities compared to the general population: a register study (IDcare) |
| **AUTHORS** | Axmon, Anna<br>Sandberg, Magnus |

## VERSION 1 – REVIEW

| | |
|---|---|
| **REVIEWER NAME** | Kraus de Camargo, Olaf |
| **REVIEWER AFFILIATION** | McMaster University, CanChild Centre for Childhood-onset Disability Research, Department of Pediatrics |
| **REVIEWER CONFLICT OF INTEREST** | No Conflict of Interest |
| **AI DISCLOSURE** | I used Claude AI in incognito chat, model Sonnet 4.6. to clarify usage of the term "risk" in statistics and in clinical settings as it pertains to this paper. |
| **DATE REVIEW RETURNED** | 08-Apr-2026 |

| | |
|---|---|
| **GENERAL COMMENTS** | Thank you for the opportunity to review this manuscript. I appreciate the benefits a registry as the one described in Sweden offers to perform such types os analysis.<br>To strengthen further your message, I would like to suggest to elaborate a bit more in the introduction about healthcare utilization and its determinants and then frame your findings accordingly. The sentence in lines 61 and 62 on page 3 describes your central hypothesis or question that you are trying to answer with the data you have at disposal. If I understood correctly, due to the almost 100% utilization of planned child health visits, that the access to healthcare services in Sweden pre-pandemic did not present significant barriers. A change in the utilization pattern could be due to the healthcare services being less accessible or the parents avoiding using them in the same way as before the pandemic. As the majority of the readers of this journal are clinicians and using the term "risk", although statistically correct, suggests a probability of an adverse health event. Maybe consider rephrasing "the risk of being hospitalized" as "healthcare utilization ratio"?<br>Two more specific suggestions are listed below:<br>1. Page 2, Line 45 - Many neurodevelopmental conditions can present with Intellectual Disability but Intellectual Disability can also be a consequence of other conditions such as traumatic brain injury, infections of the CNS and brain tumors. My suggestion is to delete the neurodevelopmental disorder part unless you accessed the registry specifically for this term.<br>2 - Page 10, Line 225 - consider adding a reference related to the lack of access to services for young adults with intellectual disabilities or childhood-onset disabilities. |

## VERSION 1 – AUTHOR RESPONSE

To strengthen further your message, I would like to suggest elaborating a bit more in the introduction about healthcare utilization and its determinants and then frame your findings accordingly.

- We have added an introduction about healthcare utilization and its determinants including a reference to the Anderson model in the introduction. We have also included reasoning based on this model in the interpretation of the results in the discussion.

The sentence in lines 61 and 62 on page 3 describes your central hypothesis or question that you are trying to answer with the data you have at disposal.

- We have removed this sentence and instead elaborated on the hypothesis and the aim of the study.

If I understood correctly, due to the almost 100% utilization of planned child health visits, that the access to healthcare services in Sweden pre-pandemic did not present significant barriers.

- We realise that this paragraph may sound counterintuitive given the hypothesis and aim of the paper. We have therefore clarified in the introduction that high access is not always equal to having all healthcare needs met.

A change in the utilization pattern could be due to the healthcare services being less accessible or the parents avoiding using them in the same way as before the pandemic.

- We are now acknowledging this in the discussion.

As the majority of the readers of this journal are clinicians and using the term "risk", although statistically correct, suggests a probability of an adverse health event. Maybe consider rephrasing "the risk of being hospitalized" as "healthcare utilization ratio"?

- We have removed all instances of “risk” (except for when it refers to the relative risk estimated by the Poisson regression).

Two more specific suggestions are listed below:

1. Page 2, Line 45 - Many neurodevelopmental conditions can present with Intellectual Disability but Intellectual Disability can also be a consequence of other conditions such as traumatic brain injury, infections of the CNS and brain tumors. My suggestion is to delete the neurodevelopmental disorder part unless you accessed the registry specifically for this term.

- We have removed this wording.

2 - Page 10, Line 225 - consider adding a reference related to the lack of access to services for young adults with intellectual disabilities or childhood-onset disabilities.

## VERSION 2 – REVIEW

| | |
|---|---|
| **REVIEWER NAME** | Kraus de Camargo, Olaf |
| **REVIEWER AFFILIATION** | McMaster University, CanChild Centre for Childhood-onset Disability Research, Department of Pediatrics |
| **REVIEWER CONFLICT OF INTEREST** | No Conflict of Interest |
| **AI DISCLOSURE** | No, I have not used AI |
| **DATE REVIEW RETURNED** | 23-Apr-2026 |

| | |
|---|---|
| **GENERAL COMMENTS** | Thank you for addressing the suggestions |
